# Supplementary material for: Content-rich biological network constructed by mining PubMed abstracts
Source: BMC Bioinformatics. 2004 Oct 8;5:147. doi: 10.1186/1471-2105-5-147 (PMC528731; doi:10.1186/1471-2105-5-147)
Supplement: Additional File 2 — The original results of the above study (non-essential files are deleted to keep the file size under the limit set by BMC bioinformatics). [file 1471-2105-5-147-S2.bz2 › chilibotAdditionalFile2/dip20/765ID9732274E18154/html/PNP_RHLB.html]

 


 **PNP** and **RHLB** 
  
Found 13 abstracts in PubMed, retrieved 13.  
 

 What does Google say? 
 PDF only 
| .edu only 

---

**Interactive relationship** (e.g. stimulation, inhibition, etc)

**Neutral relationship**- BIAcore analysis revealed real time, direct binding for bimolecular interactions between Rh1B units and for the  **RhlB**  interaction with PNPase  [ **PNP** ] .  Ref: 12181321 J Biol Chem, 2002
- Earlier work has shown that the C terminal region of RNase E is a scaffold for the binding of degradosome components and has identified specific RNase E segments necessary for its interaction with polynucleotide phosphorylase  [ **PNP** ]  PNPase  [ **PNP** ] ,  **RhlB**  RNA helicase, and enolase.  Ref: 11134527 Proc Natl Acad Sci U S A, 2001
- We also show that the C terminal half of E. coli RNase E is both sufficient and necessary for its physical interaction with the 3 5 exoribonuclease polynucleotide phosphorylase  [ **PNP** ] , the  **RhlB**  helicase, and the glycolytic enzyme enolase, which are components of a degradosome complex.  Ref: 9751718 Proc Natl Acad Sci U S A, 1998
- DEAD box  **RhlB**  RNA helicase physically associates with exoribonuclease PNPase  [ **PNP** ]  to degrade double stranded RNA independent of the degradosome assembling region of RNase E.  Ref: 12181321 J Biol Chem, 2002
- These results suggest that  **RhlB**  acts by unwinding RNA structures that impede the processive activity of PNPase  [ **PNP** ] .  Ref: 8610017 Nature, 1996
- RNase E is a large multidomain protein with N terminal ribonucleolytic activity, an RNA binding domain and a C terminal scaffold that binds PNPase  [ **PNP** ] , enolase and  **RhlB** .  Ref: 12035760 Biochem Soc Trans, 2002
- Moreover, both ATP and a non hydrolyzable analog, ATPgammaS, support the PAP I and PNPase  [ **PNP** ]  dependent degradation of the 147 residue intermediate implying that ATPase activity, such as that which may reside in  **RhlB** , a putative RNA helicase, is not necessarily required.  Ref: 9642084 J Mol Biol, 1998
- **RhlB**  in vitro can facilitate the degradation of structured RNA by PNPase  [ **PNP** ] .  Ref: 12035760 Biochem Soc Trans, 2002

**Non-interactive relationship** (e.g. studied together, co-existance, homology, etc.)

- The most important recent developments have been the discovery that the degradosome a multiprotein complex containing an endoribonuclease RNase E, an exoribonuclease polynucleotide phosphorylase  [ **PNP** ] , and a DEAD box helicase  **RhlB**  has a central role in mRNA degradation.  Ref: 10684583 Bioessays, 2000
- The Escherichia coli degradosome is a multienzyme complex with four major protein components the endoribonuclease RNase E, the exoribonuclease PNPase  [ **PNP** ] , the RNA helicase  **RhlB**  and enolase.  Ref: 9383162 Mol Microbiol, 1997
- As expected, purified degradosomes, a multi protein complex containing, among others, RNase E, PNPase  [ **PNP** ] , and  **RhlB** , generate an authentic 147 residue RNase E cleavage product from the rpsT mRNA in vitro.  Ref: 9642084 J Mol Biol, 1998
- Using an E. coli two hybrid system, together with BIAcore apparatus, we investigated the ability of three proteins, polynucleotide phosphorylase  [ **PNP** ]  PNPase  [ **PNP** ] ,  **RhlB**  RNA helicase, and enolase, a glycolytic protein, to interact physically and functionally independently of RNase E.  Ref: 12181321 J Biol Chem, 2002
- However, binding of  **RhlB**  or PNPase  [ **PNP** ]  to enolase was not detected under the same conditions.  Ref: 12181321 J Biol Chem, 2002
- Two other components are a 3 exoribonuclease polynucleotide phosphorylase  [ **PNP** ] , PNPase  [ **PNP** ]  and a DEAD box RNA helicase RNA helicase B,  **RhlB** .  Ref: 12035760 Biochem Soc Trans, 2002
- These results suggest that MrsC, directly or indirectly, controls endonucleolytic processing of mRNAs that may be independent of the RNase E PNPase  [ **PNP** ]   **RhlB**  multiprotein complex.  Ref: 9537393 J Bacteriol, 1998
- RNA molecules with 3 ends incorporated into a stable stem loop structure could not readily be degraded by purified polynucleotide phosphorylase  [ **PNP** ]  or by the degradosome, even though the degradosome contains active  **RhlB**  helicase which normally facilitates degradation of structured RNA.  Ref: 9933592 J Biol Chem, 1999
- Furthermore, in the absence of RNase E, purified  **RhlB**  can carry out ATP dependent unwinding of double stranded RNA and consequently modulate degradation of double stranded RNA together with the exonuclease activity of PNPase  [ **PNP** ] .  Ref: 12181321 J Biol Chem, 2002
- Our results demonstrate that a degradosome like complex containing RNase E, PNPase  [ **PNP** ] , and  **RhlB**  can form spontaneously in vitro in the absence of all other cellular components.  Ref: 10521403 Genes Dev, 1999
- These results provide evidence for the first time that both functional and physical interactions of individual degradosome protein components can occur in the absence of RNase E and raise the prospect that the RNase E independent complexes of  **RhlB**  RNA helicase and PNPase  [ **PNP** ] , detected in vivo, may constitute mini machines that assist in the degradation of duplex RNA in structures physically distinct from multicomponent RNA degradosomes.  Ref: 12181321 J Biol Chem, 2002
- Our results demonstrate that the carboxy terminal half CTH of ribonuclease E RNase E contains the binding sites for the three other major degradosomal components, the DEAD box RNA helicase  **RhlB** , enolase, and polynucleotide phosphorylase  [ **PNP** ]  PNPase  [ **PNP** ] .  Ref: 9732274 Genes Dev, 1998
